# Production of knockout mice by DNA microinjection of various CRISPR/Cas9 vectors into freeze-thawed fertilized oocytes

**DOI:** 10.1186/s12896-015-0144-x

**Published:** 2015-05-22

**Authors:** Yoshiko Nakagawa, Tetsushi Sakuma, Takuya Sakamoto, Masaki Ohmuraya, Naomi Nakagata, Takashi Yamamoto

**Affiliations:** Center for Animal Resources and Development, Kumamoto University, Kumamoto, 860-0811 Japan; Department of Mathematical and Life Sciences, Graduate School of Science, Hiroshima University, Hiroshima, 739-8526 Japan

**Keywords:** Knockout mouse, Pronuclear microinjection, *In vitro* fertilization, Freeze-thawing, CRISPR/Cas9, Double-nicking, FokI-dCas9

## Abstract

**Background:**

Clustered regulatory interspaced short palindromic repeats (CRISPR)/CRISPR-associated protein 9 (Cas9)-mediated genome editing permits the rapid production of genetically engineered mice. To make the most of this innovative technology, a streamlined procedure is needed for the robust construction of CRISPR/Cas9 vectors, the efficient preparation of mouse oocytes, and refined genotyping strategies. Although we previously demonstrated the applicability of oocyte cryopreservation technologies and various genotyping methods in the production of transcription activator-like effector nuclease-mediated genome editing in mice, it has not yet been clarified whether these techniques can be applied to the CRISPR/Cas9-mediated generation of knockout mice. In this study, we investigated easy, efficient, and robust methods of creating knockout mice using several CRISPR/Cas9 systems.

**Results:**

We constructed three types of CRISPR/Cas9 vectors expressing: 1) single guide RNA (gRNA) and Cas9 nuclease, 2) two gRNAs and Cas9 nickase, and 3) two gRNAs and FokI-dCas9, targeting the same genomic locus. These vectors were directly microinjected into the pronucleus of freeze-thawed fertilized oocytes, and surviving oocytes were transferred to pseudopregnant ICR mice. Cas9 nuclease resulted in the highest mutation rates with the lowest birth rates, while Cas9 nickase resulted in the highest birth rates with the lowest mutation rates. FokI-dCas9 presented well-balanced mutation and birth rates. Furthermore, we constructed a single all-in-one FokI-dCas9 vector targeting two different genomic loci, and validated its efficacy by blastocyst analysis, resulting in highly efficient simultaneous targeted mutagenesis.

**Conclusions:**

Our report offers several choices of researcher-friendly consolidated procedures for making CRISPR/Cas9-mediated knockout mice, with sophisticated construction systems for various types of CRISPR vectors, convenient preparation of *in vitro* fertilized or mated freeze-thawed oocytes, and an efficient method of mutant screening.

**Electronic supplementary material:**

The online version of this article (doi:10.1186/s12896-015-0144-x) contains supplementary material, which is available to authorized users.

## Background

Genetically engineered mice (GEM) have played an essential part in elucidating the functions of specific genes, the mechanisms of disease, embryogenesis and differentiation over recent decades. Although massive numbers of GEM have been generated and analyzed throughout the world [1], there is still a demand for more efficient methods of generating *de novo* GEM.

Genome editing using programmable nucleases, such as zinc-finger nucleases (ZFNs), transcription activator-like effector nucleases (TALENs), and clustered regularly interspaced short palindromic repeats (CRISPR)/ CRISPR-associated protein 9 (Cas9), is an easy and efficient strategy to generate GEM [2]. In particular, CRISPR/Cas9 provides the most convenient method by which to produce GEM because of its simple construction and high DNA double-strand break (DSB)-inducing activity [3]. A conventional CRISPR/Cas9 system consists of Cas9 nuclease and a single guide RNA (gRNA) that targets a specified genomic locus containing a 20-base sequence defined by the gRNA and a protospacer adjacent motif (PAM) sequence defined by Cas9, the complex then cleaves the double-stranded DNA. DSBs are mainly repaired by error-prone non-homologous end-joining (NHEJ), randomly inducing insertions and/or deletions, which cause targeted gene disruption.

The conventional CRISPR/Cas9 complex does not form a dimer, unlike ZFNs and TALENs, and it often leads to off-target mutations with high frequency [4-6]. Although the frequencies of off-target mutations in animal embryos, especially in mice and rats, are reportedly not so high [7,8], they do exist and therefore present a potential risk even in animals. To improve the specificity, two derivative technologies have been developed; double-nicking using Cas9 nickase [9-13] and FokI-dimerization using nuclease-deficient Cas9 fused to FokI (FokI-dCas9) [14,15]. In both strategies, paired gRNAs are used to recruit two molecules of Cas9 nickase or FokI-dCas9 on juxtaposed positions of the target genomic locus, resulting in DNA cleavage. Importantly, single gRNA-guided Cas9 nickase can only induce a nick, which is less mutagenic than a DSB. Furthermore, single gRNA-guided FokI-dCas9 does not damage the genomic DNA at all. Therefore, off-target mutations are significantly reduced in these two derivative strategies.

To generate knockout mice using the CRISPR/Cas9 system, Cas9 mRNA and gRNA are generally synthesized using *in vitro* transcription and used for microinjection [16-21]. However, Mashiko and colleagues described a simplified protocol using pronuclear microinjection of circular plasmid expressing Cas9 and a gRNA [7,22]. Direct use of plasmids avoids the need for laborious *in vitro* transcription and purification steps, and plasmids offer high stability compared with RNAs enabling the robust production of knockout mice. In addition, we recently established an all-in-one CRISPR/Cas9 vector system for the assembly of multiple gRNA cassettes and a Cas9 nuclease/nickase cassette in a single vector [23]. When combined with these procedures, efficient production of knockout mice mediated by paired gRNA-guided CRISPR systems has been thought to be achieved.

Regarding the preparation of oocytes for microinjection to generate knockout mice with genome editing technologies, fertilized oocytes were usually collected by mating a male mouse and a superovulated female mouse. However, preparing fresh fertilized oocytes for every experiment is time-consuming and laborious work. We have previously established a procedure of using freeze-thawed fertilized oocytes created by *in vitro* fertilization (IVF) for the TALEN-mediated generation of knockout mice [24], as well as previous reports of transgenic mouse production [25,26]. In addition, Li and colleagues recently reported that fresh fertilized oocytes created by IVF could be used for conventional CRISPR/Cas9-mediated mouse genome editing [21]. However, Han and colleagues reported that they failed to produce genome edited mice by pronuclear injection of CRISPR/Cas9 plasmid, while cytoplasmic injection of Cas9 mRNA and purified gRNA resulted in successful genome editing [27]. Thus, the applicability of cryopreservation and IVF technologies for various CRISPR systems is another important advance in establishing the streamlined CRISPR/Cas9 plasmid injection-mediated production of knockout mice.

Here, we expand the multiplex gRNA assembly system to include FokI-dCas9, and construct Cas9 nuclease-, Cas9 nickase- and FokI-dCas9-type vectors for the generation of knockout mice. The gRNAs for each system are designed to target the same locus of an endogenous gene, and comparisons of the birth rate and the mutation rate are conducted. The applicability of cryopreservation and IVF is also investigated. We further examined whether pronuclear injection of a single all-in-one plasmid expressing FokI-dCas9 and four gRNAs could achieve multiplex genome editing in mouse embryos.

## Results and discussion

### Construction of an all-in-one vector system for FokI-dCas9-mediated genome editing

**Figure 1:**
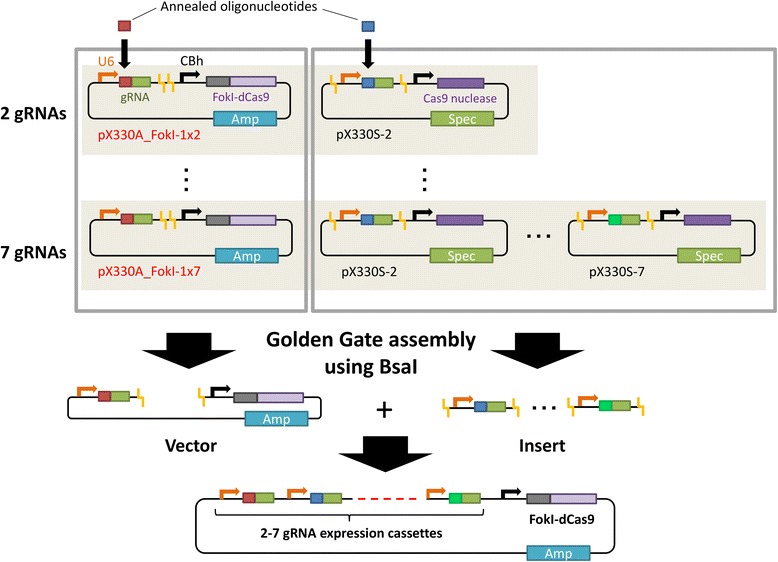
Schematic illustration of the all-in-one FokI-dCas9 vector construction system. Single gRNA-expressing vectors are constructed by the insertion of annealed oligonucleotides. Subsequently, the gRNA cassettes are assembled using Golden Gate cloning. Amp, ampicillin; Spec, spectinomycin; U6, human U6 promoter; CBh, chicken beta-actin short promoter.

We previously established a system for creating all-in-one CRISPR/Cas9 vectors expressing Cas9 nuclease or nickase and up to seven gRNAs [23], and have distributed these plasmids as a “Multiplex CRISPR/Cas9 Assembly System Kit” via Addgene (Kit #1000000055). To expand the system for FokI-dCas9-mediated genome editing, we first created pX330A_FokI-1x(2–7) vectors. Using these newly constructed plasmids and pX330s-(2–7) vectors included in the Addgene Kit, we can create an all-in-one vector for FokI-dCas9-mediated genome editing, not only for single but also for multiplex gene editing (Figure 1). The new vectors will expectedly be distributed by Addgene as a supplemental package of the current kit (Multiplex CRISPR dCas9/FokI-dCas9 Accessory Pack).

### Design of CRISPR/Cas9 nuclease, Cas9 nickase, and FokI-dCas9 vectors to target the interleukin-11 (*IL11*) gene

**Figure 2:**
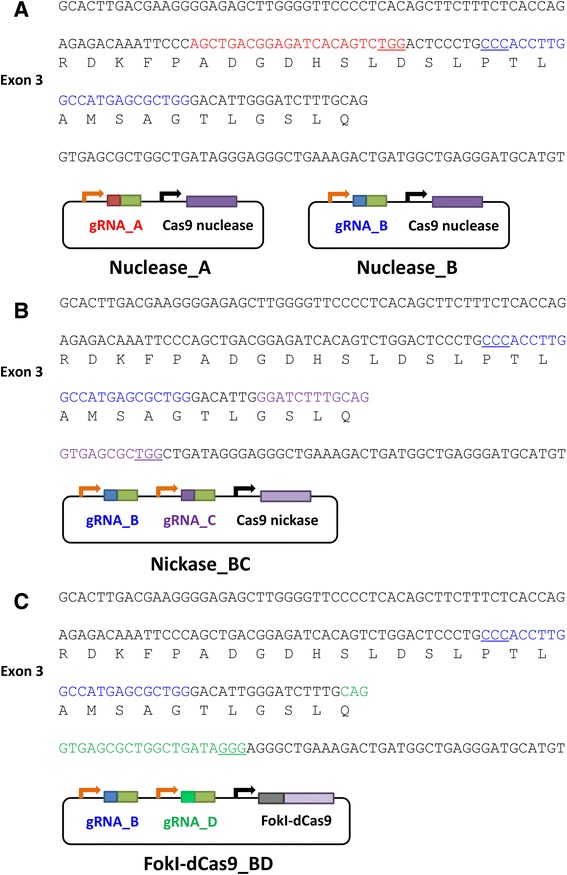
The target sequence and the constructed vectors. The genomic region around exon 3 of the *IL11* gene was targeted by Cas9 nuclease **(A)**, Cas9 nickase **(B)**, or FokI-dCas9 **(C)** vectors. The target sequence of each gRNA is indicated by colored bases. The PAM sequence is underlined.

To compare the applicability of the three CRISPR/Cas9 systems with Cas9 nuclease, Cas9 nickase, or FokI-dCas9 in mouse genome editing, we designed and constructed CRISPR/Cas9 vectors as shown in Figure 2. Although the three systems require different design parameters and it is difficult to compare the genome editing efficacy with exactly the same gRNAs, we tried to target almost the same genomic region, which is around exon 3 of the *IL11* gene. For Cas9 nuclease, two gRNAs, gRNA_A and gRNA_B, were designed within the exon and oligonucleotides for these gRNAs were cloned separately (Figure 2A; Nuclease_A and Nuclease_B vectors). For Cas9 nickase, gRNA_B and gRNA_C were designed with a 7-bp offset (Figure 2B: Nickase_BC vector). When inducing double nick-mediated genome modification, the optimal range of the offset length is around 0–10 bp [23], whereas FokI-dCas9 requires 13- to 18-bp offsets for highly-efficient targeted mutagenesis [14]. Thus, we designed another gRNA, gRNA_D, in combination with gRNA_B for FokI-dCas9 (Figure 2C; FokI-dCas9_BD vector).

### Activity validation of the all-in-one FokI-dCas9 vector

**Figure 3:**
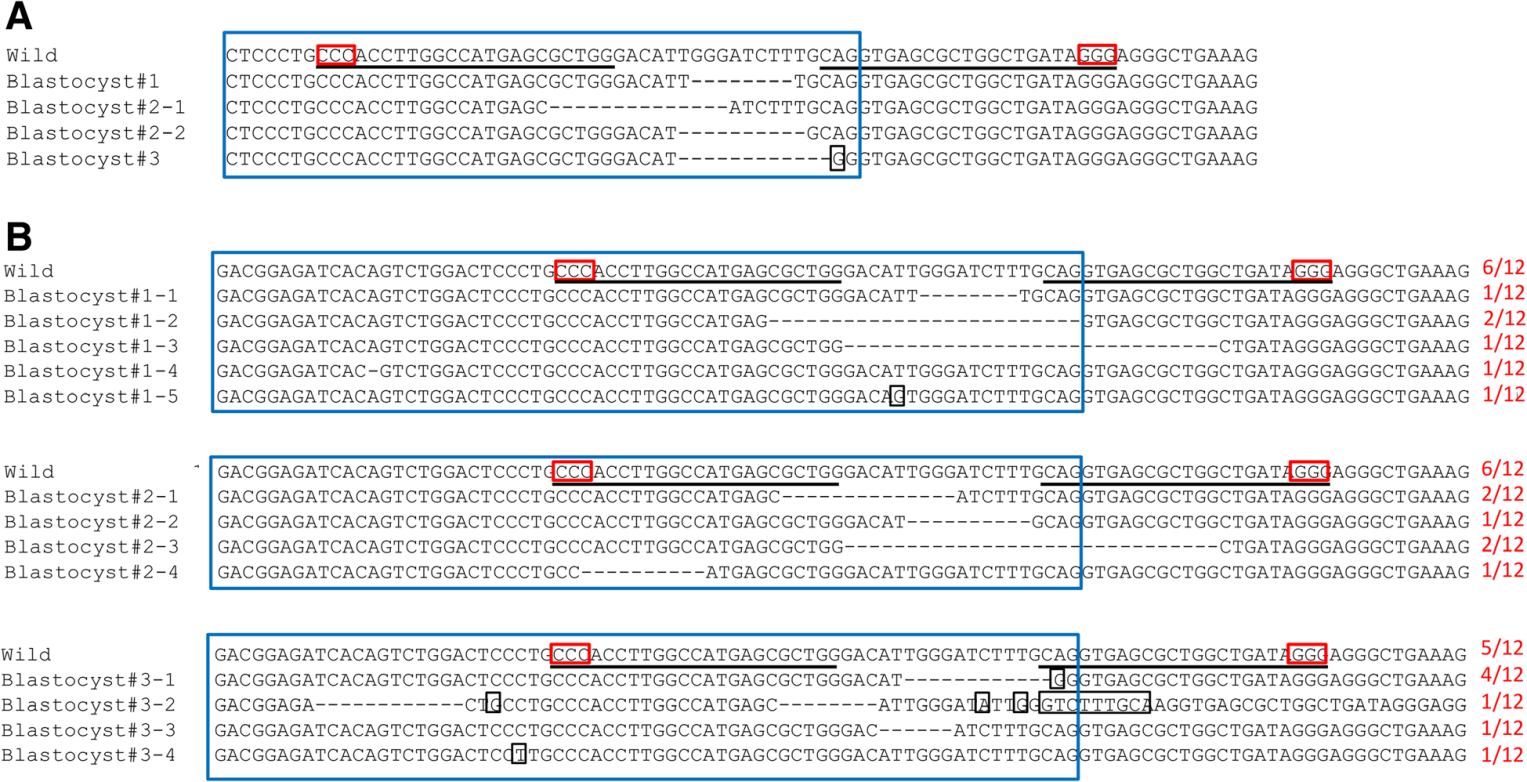
Sequence analysis of blastocysts injected with the FokI-dCas9_BD vector. The PCR products were analyzed by direct sequencing **(A)**, followed by sequencing of subcloned plasmids **(B)**. The wild-type *IL11* sequence is shown at the top (Wild) with the gRNA target sequences (underlined). The PAM sequence is enclosed in red boxes. Deletions are indicated by dashes and substitutions and insertions are enclosed in a black box. A blue box indicates the partial sequence of exon 3. Only one or two types of mutations were detected in direct sequencing **(A)**, whereas various mutation patterns were observed in subcloned sequencing **(B)**. Numbers in red letters on the right side of each sequence indicate the frequencies of the corresponding alleles (number of the allele/number of total clones).

Since the genome editing potential of the all-in-one vector containing the FokI-dCas9 cassette was unknown, we evaluated the activity of the FokI-dCas9_BD vector in mouse embryos. The FokI-dCas9_BD vector was microinjected into the pronucleus of mouse fertilized oocytes. Twenty-one survival oocytes were cultured for 3.5 days. We observed seven blastocysts, and six of these embryos developed into expanded blastocysts. Each blastocyst was collected into a microtube, and then PCR was performed to amplify the targeted locus. We verified that at least one mutation had been induced in three embryos by direct sequencing (Figure 3A). In addition, bacterial cloning of the PCR products followed by DNA sequencing revealed high mutation rates (≥50%) (Figure 3B), confirming the powerful potential of the all-in-one FokI-dCas9 vector for GEM generation.

### GEM generation by microinjection of all-in-one CRISPR/Cas9 plasmids into freeze-thawed fertilized oocytes

We next conducted microinjection of the three types of CRISPR/Cas9 vectors into freeze-thawed fertilized oocytes for efficient and convenient genome engineering in mice. Using reproductive engineering techniques, such as IVF and freeze-thawing, fertilized oocytes could be obtained with high efficiency (Additional file 1). Each CRISPR/Cas9 vector was directly injected into the pronucleus of freeze-thawed fertilized oocytes prepared by mating or IVF. The survival oocytes after microinjection were transferred into the oviducts of pseudopregnant ICR female mice. All the surrogate female mice gave birth to pups (Table 1), and all the pups excluding one pup died from cannibalism were genotyped by direct sequencing analysis (Table 1 and Additional file 2). Although we have shown that the combinatorial screening strategy is required for complete genotyping [28], direct sequencing is reportedly able to screen mutant mice with high mutation rates [7].

**Table 1:** **Generation of** ***IL11*** **mutants using freeze-thawed fertilized oocytes**

**Method**	**Vector**	**Injected**	**Survived**	**Transferred**	**Pups**	**Analyzed**	**Mutants**
Mating	Nuclease_A	76	54 (71.1)	54	4 (7.4)	4	4 (100)
	Nuclease_B	72	55 (76.4)	55	5 (9.1)	5	4 (80.0)
	Nickase_BC	108	94 (87.0)	94	18 (19.1)	17	1 (5.9)
	FokI-dCas9_BD	55	46 (83.6)	33	6 (18.2)	6	4 (66.6)
	FokI-dCas9_BD	58*	55 (94.8)	34	13 (38.2)	13	11 (84.6)
IVF	Nuclease_A	32	25 (78.1)	25	3 (12.0)	3	3 (100)
	Nuclease_B	34	22 (64.7)	22	2 (9.1)	2	2 (100)
	Nickase_BC	32	27 (84.4)	27	6 (22.2)	6	0 (0)
	FokI-dCas9_BD	39	35 (89.7)	34	6 (17.6)	6	2 (33.3)

Numbers in brackets represent percentages.

*Fertilized oocytes were collected from females at 8 weeks of age, whereas 5-week-old females were used for the other injections that oocytes were fertilized by mating.

Sequencing analysis revealed that all three types of CRISPR/Cas9 vectors could produce mutant mice, but the birth rates and mutation rates of each method were not comparable. In the Cas9 nuclease group, the birth rates were relatively low (7.4–12.0%), whereas the mutation rates were high (80–100%). Conversely, more pups were born in the Cas9 nickase group (19.1% and 22.2%), while the number of mutants were fewer than in the Cas9 nuclease group (5.9% and 0%). Interestingly, the FokI-dCas9 group exhibited moderate birth rates (18.2% and 17.6%) and mutation rates (66.6% and 33.3%). To confirm the superiority of FokI-dCas9-mediated method, we additionally injected the FokI-dCas9_BD vector into freeze-thawed fertilized oocytes collected from mature female mice (8 weeks of age) mated with male mice. Consistent with the results using the oocytes collected from immature female mice (5 weeks of age), the birth rate and the mutation rate were sufficiently high (38.2% and 84.6%, respectively). These results suggest not only the robustness of FokI-dCas9-mediated mouse genome editing, but also the applicability of oocytes from female mice at various weeks of age, as shown previously using TALENs [24]. Although these results do not necessarily reflect the general properties of the three CRISPR/Cas9 systems, they represent an important practical example of CRISPR/Cas9-mediated GEM generation. Importantly, the low birth rates of Cas9 nuclease plasmid-injected mice are comparable with previous studies using inbred strains [29,30], although high birth rates can be achieved using B6D2F1 (BDF1) hybrid mice [7]. We thus concluded that FokI-dCas9 plasmid injection might be a powerful strategy for generating knockout mice, especially for inbred strains.

### Transgene analysis and off-target analysis

Since plasmid DNA was used for microinjection, we investigated whether DNA vectors were integrated into the genome. Genomic PCR of the coding sequence of Cas9 variants and FokI was performed to detect genomic integrants. In the two founders generated by the injection of Nuclease_B vector, the Cas9 gene fragment was amplified (Additional file 3). Altogether, 10% (2/20) of the mutant mice carried the transgene. Although the observed transgenicity is slightly higher than in Mashiko’s report (4.3%) [7], this may not be a major issue because the transgene can be removed by mating before expansion of the mutant strain.

Finally, potential off-target sites for each gRNA target site were analyzed by direct sequencing. The top three candidates for each site were selected using the CRISPR design tool (http://crispr.mit.edu/) (Additional file 4). We then sequenced all of the 20 founders (seven founders for Nuclease_A, six founders for Nuclease_B, one founder for Nickase_BC, and six founders for FokI-dCas9_BD), but no off-target mutations were detected. However, our analysis and all of the previously reported off-target analyses in mice provide limited genomic context, and the potential risk of off-target mutagenesis in disparate parts of the genome is undeniable. In fact, Tsai and colleagues recently revealed that CRISPR/Cas9 can induce various genomic rearrangements including megabase-scale large deletions and chromosomal translocations in cultured cells [31]. They also showed that most of these experimentally demonstrated off-target sites were not identified as off-target candidates using any computational prediction tool including the CRISPR design tool. Also in mice, these concealed off-target effects can exist, but they are difficult to detect because they cannot be predicted by *in silico* analysis. We thus believe that a highly specific gene targeting strategy, such as FokI-dCas9, is important not only for cultured cell applications but also for the creation of GEM.

### Multiplex genome editing in mouse embryos using a single all-in-one FokI-dCas9 vector

**Figure 4:**
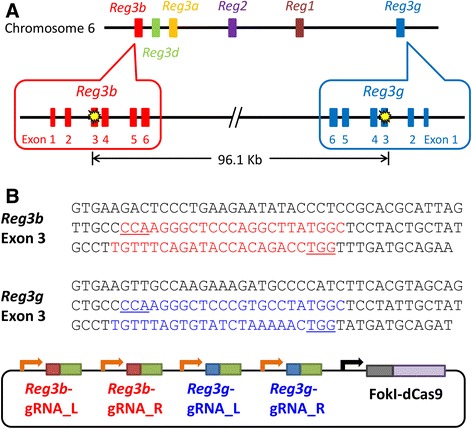
Schematic design of multiple gene targeting using all-in-one FokI-dCas9 vector. **(A)** The genomic context around the targeted loci. The target sites are located in exon 3 of *Reg3b* and *Reg3g* genes. **(B)** The target sequence and the constructed vectors. The target sequence of each gRNA is indicated by colored bases. The PAM sequence is underlined.

**Figure 5:**
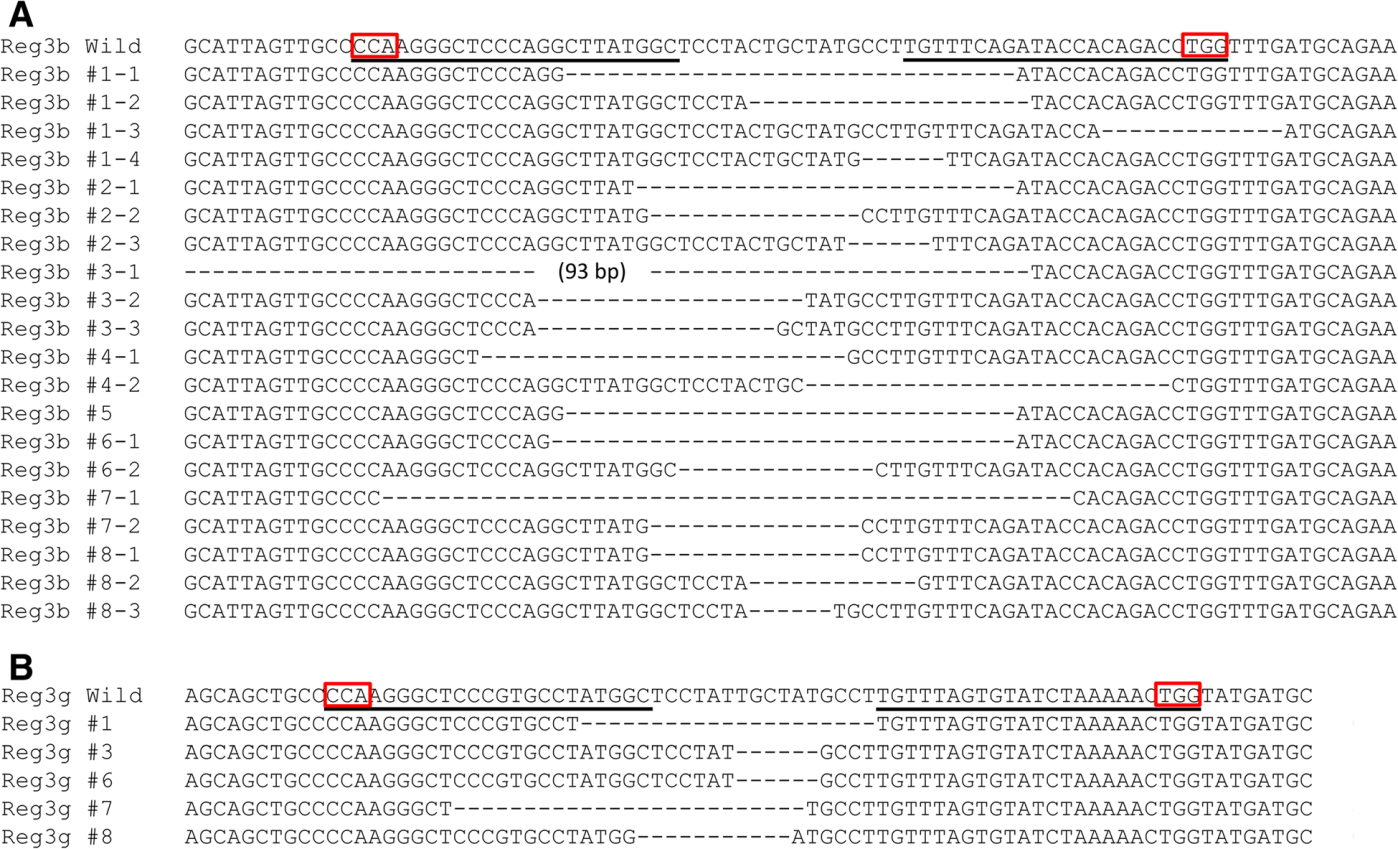
Sequence analysis of blastocysts injected with all-in-one FokI-dCas9 vector simultaneously targeting *Reg3b* and *Reg3g* genes. The PCR products were analyzed by direct sequencing to identify mutations in *Reg3b* **(A)** and *Reg3g* **(B)** loci. The wild-type sequence of *Reg3b* and *Reg3g* are shown at the top (Wild) with the gRNA target sequences (underlined). The PAM sequence is enclosed in red boxes. Deletions are indicated by dashes. Blastocyst numbers on the left side of each sequence in **(A)** and **(B)** are identical. Most blastocysts had multiple types of mutations at the *Reg3b* locus. The wild-type *Reg3b* sequence was not detected in blastocyst #4 and #6. In the other blastocysts, the wild-type *Reg3b* sequence was detected along with mutant sequence. Regarding *Reg3g* locus, the wild-type sequence was detected in all blastocysts with mutant sequence.

We finally examined whether multiple gene targeting could be applied using microinjection of a single all-in-one FokI-dCas9 vector simultaneously expressing four gRNAs and FokI-dCas9. We designed gRNAs to target exon 3 of regenerating islet-derived 3 beta (*Reg3b*) and regenerating islet-derived 3 gamma (*Reg3g*) genes, which are located in the chromosome 6 with 96.1-kb distance (Figure 4A and B). The all-in-one FokI-dCas9 vector was microinjected into the pronucleus of fertilized oocytes. Twenty-eight survival oocytes were cultured for 3.5 days, and nine embryos developed into expanded blastocysts. Each blastocyst was collected into a microtube, and then PCR was performed to amplify around the both target loci. Direct sequencing analyses revealed that more than 50% of the embryos possessed mutated alleles in the both target loci (8/9 in *Reg3b* and 5/9 in *Reg3g*) (Figure 5A and B). More importantly, five embryos (#1, 3, 6, 7, and 8) possessed mutations in the both genes, showing high potential for creating double knockout mice mediated by microinjection of all-in-one FokI-dCas9 vector. We further performed out-out PCR to investigate whether chromosomally deleted alleles exist, but no intended amplicons were observed using blastocyst PCR genotyping. Further examinations are needed to clarify the possible existence of chromosomally deleted alleles in pups.

## Conclusions

Our study compared the genome editing efficiency in one-cell mouse embryos microinjected with various CRISPR/Cas9 vectors expressing gRNA(s) and Cas9 nuclease, Cas9 nickase, or FokI-dCas9, constructed by an all-in-one vector system. In addition, we demonstrated the applicability of IVF and freeze-thawing of oocytes in GEM generation mediated by the methods described above, which makes preparation of fertilized oocytes more convenient. Our findings suggest that the FokI-dCas9-mediated approach is a highly reliable strategy for creating GEM, including double knockout mice, and we provide accessible platforms for the CRISPR/Cas9-based generation of GEM.

## Methods

### Plasmids

All-in-one CRISPR/Cas9 vectors for Cas9 nuclease and nickase were constructed using the Multiplex CRISPR/Cas9 Assembly System Kit (Addgene, Cambridge, MA, USA; Kit #1000000055) as previously described [23]. For FokI-dCas9 vectors, a H840A mutation was introduced in pX330A_D10A vectors using inverse PCR and the In-Fusion cloning method (Clontech, Palo Alto, CA, USA) (pX330A_dCas9 vectors). Subsequently, the coding sequence of FokI was amplified by PCR from ptCMV-136/63-VR vector contained in the Platinum Gate TALEN Kit (Addgene; Kit #1000000043) [32] using the FokI-dCas9-ins-F and FokI-dCas9-ins-R primers listed in Additional file 5. The amplified fragment was cloned using the In-Fusion cloning method into pX330A_dCas9 vectors, which were amplified by inverse PCR using the FokI-dCas9-vec-F and FokI-dCas9-vec-R primers (Additional file 5) (pX330A_FokI vectors). After the construction of pX330A_FokI vectors, oligonucleotides were inserted and Golden Gate cloning was performed as previously described [23] to construct the FokI-dCas9_BD vector.

### Animals

All animal experiments were approved by the Animal Care and Experimentation Committee of the Center for Animal Resources and Development, Kumamoto University, and were carried out in accordance with the approved guidelines.

### IVF and freeze-thawing of fertilized oocytes

The IVF and freeze-thawing procedure was performed according to a previous report [24]. The cauda epididymides were obtained from C57BL/6 male mice over 12 weeks of age, and spermatozoa were suspended in FERTIUP® Mouse Sperm Preincubation Medium (Kyudo, Saga, Japan). Subsequently, the sample was incubated for 1 h at 37°C in 5% CO_2_ and 95% humidified air until insemination. C57BL/6 female mice were superovulated using 7.5 IU of pregnant mare serum gonadotropin (Serotropin; Aska Pharmaceutical, Tokyo, Japan) and 7.5 IU of human chorionic gonadotropin (Gonatropin; Aska Pharmaceutical, Tokyo, Japan) at 8–12 weeks of age, and their oviducts were transferred to fertilization dishes containing a drop of CARD MEDIUM (Kyudo, Saga, Japan). The cumulus-oocyte complexes were introduced into the drop of CARD MEDIUM. After preincubation of the fresh sperm, the sperm suspension was added to the drop containing cumulus-oocyte complexes. The fertilization dishes were incubated at 37°C in 5% CO_2_ and 95% humidified air. After 3 h, the inseminated oocytes were rinsed three times with human tubal fluid medium. After 2 h, the oocytes were cryopreserved by a simple vitrification method [33,34]. At later time points, the cryopreserved oocytes were thawed by a thawing method for vitrified oocytes using warmed PB1 containing 0.25 M sucrose [33,34]. The freeze-thawed oocytes were used for microinjection.

### Mating and freeze-thawing of fertilized oocytes

C57BL/6 female mice were induced to superovulate using pregnant mare serum gonadotropin and human chorionic gonadotropin at 5 or 8 weeks of age, and then mated with C57BL/6 male mice. The fertilized oocytes were collected from females displaying vaginal plugs. The oocytes were cryopreserved as described above. The frozen oocytes were thawed and used for microinjection.

### Single blastocyst assay

Fertilized oocytes were collected from C57BL/6 superovulated female mice mated with C57BL/6 male mice. After freeze-thawing of oocytes, FokI-dCas9 vector was diluted in DNase-free PBS at 5 ng/μl, and then microinjection was performed. Survival oocytes were cultured in potassium simplex optimized medium with amino acids (KSOM-AA) at 37°C in 5% CO_2_ and 95% humidified air for 3.5 days. For PCR of single blastocysts, 1 μl of KSOM-AA containing 1 blastocyst was transferred to the wall near the bottom of a 0.2-mL PCR tube. Blastocyst lysates were prepared by an alkaline lysis method. To detect the targeted *IL11* gene, PCR was performed using KOD FX (TOYOBO, Osaka, Japan) with the primers listed in Additional file 5 under the following conditions: 94°C for 2 min; followed by 40 cycles of 98°C for 10 s, 60°C for 30 s, and 68°C for 25 s. Each PCR product was subjected to automatic electrophoresis using MultiNA (Shimadzu Corporation, Kyoto, Japan). The PCR products were purified and analyzed by direct sequencing, and then the products harboring mutations were subcloned into pTA2 vector using a Target Clone -Plus- (TOYOBO). Sequencing was performed using an ABI 3130 Genetic Analyzer (Life Technologies, Carlsbad, CA, USA) with a BigDye Terminator v1.1 Cycle Sequencing Kit (Life Technologies). Also for *Reg3b* and *Reg3g*, PCR was performed using KOD FX with the primers listed in Additional file 5. Subsequently, automatic electrophoresis and direct sequencing were performed as described above.

### Microinjection and transfer

Each CRISPR/Cas9-plasmid DNA was diluted in DNase-free PBS at 5 ng/μl for microinjection. The plasmid DNA was injected into the pronucleus of fertilized oocytes. The injected oocytes were cultured in KSOM-AA at 37°C in 5% CO_2_ and 95% humidified air for 1 h. Surviving oocytes were transferred to the oviducts of pseudopregnant ICR female mice.

### Mutation analysis of the *IL11* gene

Tail lysates were prepared by an alkaline lysis method and PCR was performed using KOD FX and the primers listed in Additional file 5 under the following conditions: 94°C for 2 min; followed by 38 cycles of 98°C for 10 s, 60°C for 30 s, and 68°C for 25 s. Each PCR product was analyzed by direct sequencing as described above.

### Transgene analysis

To examine integration of vector DNA, we designed primers for Cas9, dCas9, and FokI. Each founder was examined the integration of Cas9 or dCas9, and FokI by genomic PCR using KOD FX and the primers listed in Additional file 5. The PCR products were analyzed by automatic electrophoresis using MultiNA.

### Off-target analysis

Candidates for off-target sequences were selected with the CRISPR Design tool (http://crispr.mit.edu/) (Additional file 3). Genomic regions around the top three potential off-target sites were amplified by PCR from founders using KOD FX, the primers used are listed in Additional file 5.

## Additional files

Additional file 1:
**Summary of the results of freeze-thawing in C57BL/6 mice.**
Additional file 2:
**Mutation analysis of PCR products from founders.** Sequence analysis of PCR products from founders injected with Nuclease_A (A), Nuclease_B (B), Nickase_BC (C), and FokI-dCas9_BD (D,E) vectors. Founder numbers are shown on the left side. IA, IB, and IBD were generated from oocytes fertilized by IVF. MA, MB, MBC, and MBD were generated from oocytes fertilized by mating. 8W were generated from oocytes of mature females by mating. The wild-type *IL11* sequence is shown at the top (Wild) with the gRNA target sequences (underlined). The PAM sequence is enclosed in a red box. Deletions are indicated by dashes, and insertions or substitutions are enclosed in a black box. The exon 3 sequence is enclosed in a blue box. Additional file 3:
**Transgene analysis of founders.** (A) Schematic drawing of the primers for each Cas9 variant. Red bars indicate primers for FokI detection. Blue bars indicate primers for Cas9 or dCas9 detection. The lengths of PCR products for FokI and Cas9/dCas9 are 395 and 473 bp, respectively. (B) Pseudo-gel images of transgene analysis. When vector DNA was integrated, a band of approximately 473-bp or 395-bp appears. Founder numbers shown on the upper side of each image are identical to Additional file 2. Red circles indicate transgene-positive founders. A P, B P, BC P, and BD P indicate positive controls for PCR amplification with Nulcease_A, Nuclease_B, Nickase_BC, and FokI-dCas9_BD plasmids, respectively, as templates. M, molecular weight maker (pGEM DNA Markers; Promega, Madison. WI, USA). W, negative control using founders without vector injection. N, negative control without template DNA. Additional file 4:
**Top three candidates for potential off-target sites (5’-3’).**
Additional file 5:
**Primer sets for PCR.**

## Abbreviations

CRISPR: Clustered regulatory interspaced short palindromic repeats
Cas9: CRISPR-associated protein 9
gRNA: Guide RNA
GEM: Genetically engineered mice
ZFN: Zinc-finger nuclease
TALEN: Transcription activator-like effector nuclease
DSB: DNA double-strand break
PAM: Protospacer adjacent motif
NHEJ: Non-homologous end-joining
IVF: *In vitro* fertilization
*IL11*: Interleukin-11
*Reg3b*: Regenerating islet-derived 3 beta
*Reg3g*: Regenerating islet-derived 3 gamma
KSOM-AA: Potassium simplex optimized medium with amino acids
