# Supplementary material for: Production of knockout mice by DNA microinjection of various CRISPR/Cas9 vectors into freeze-thawed fertilized oocytes
Source: BMC Biotechnol. 2015 May 22;15:33. doi: 10.1186/s12896-015-0144-x (PMC4440308; doi:10.1186/s12896-015-0144-x)
Supplement: Additional file 1: — Summary of the results of freeze-thawing in C57BL/6 mice. [file 12896_2015_144_MOESM1_ESM.docx]

**Additional file 1.** **Summary of the results of freeze-thawing in C57BL/6 mice**

| Method | Females | Collected oocytes | Frozen oocytes | Recovered oocytes | Normal oocytes | Fertility |
| --- | --- | --- | --- | --- | --- | --- |
| Mating | 32 | 721 | 334 | 317 (94.9%) | 286 (90.2%) | 334/721 (46.3%) |
| IVF | 13 | 294 | 294 | 285 (96.9%) | 258 (90.5%) | 235/258 (91.1%) |
