# Supplementary figures and images for: Production of knockout mice by DNA microinjection of various CRISPR/Cas9 vectors into freeze-thawed fertilized oocytes
Source: BMC Biotechnol. 2015 May 22;15:33. doi: 10.1186/s12896-015-0144-x (PMC4440308; doi:10.1186/s12896-015-0144-x)

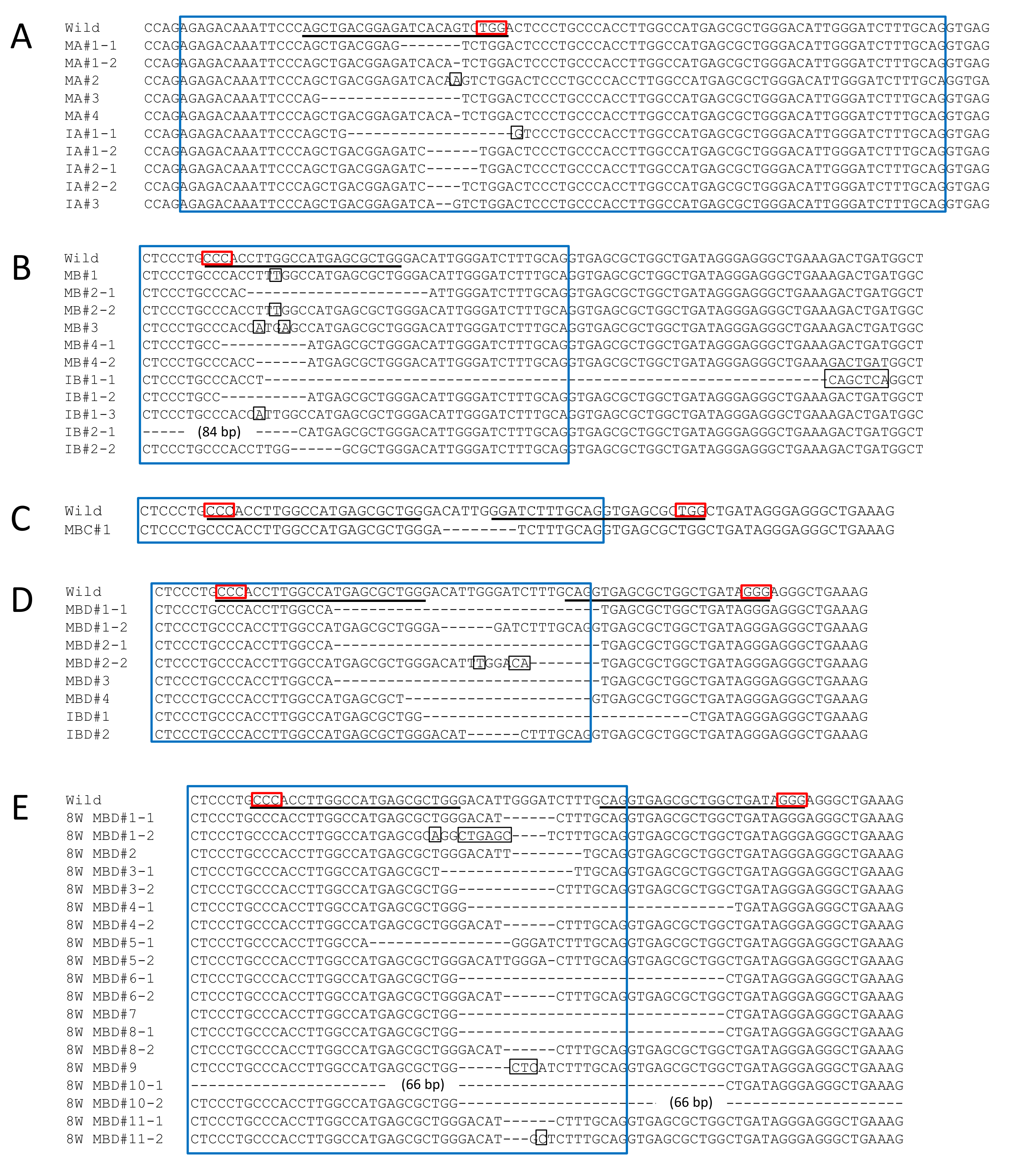

Supplement: Additional file 2: — Mutation analysis of PCR products from founders. Sequence analysis of PCR products from founders injected with Nuclease_A (A), Nuclease_B (B), Nickase_BC (C), and FokI-dCas9_BD (D,E) vectors. Founder numbers are shown on the left side. IA, IB, and IBD were generated from oocytes fertilized by IVF. MA, MB, MBC, and MBD were generated from oocytes fertilized by mating. 8W were generated from oocytes of mature females by mating. The wild-type IL11 sequence is shown at the top (Wild) with the gRNA target sequences (underlined). The PAM sequence is enclosed in a red box. Deletions are indicated by dashes, and insertions or substitutions are enclosed in a black box. The exon 3 sequence is enclosed in a blue box. [file 12896_2015_144_MOESM2_ESM.tiff]

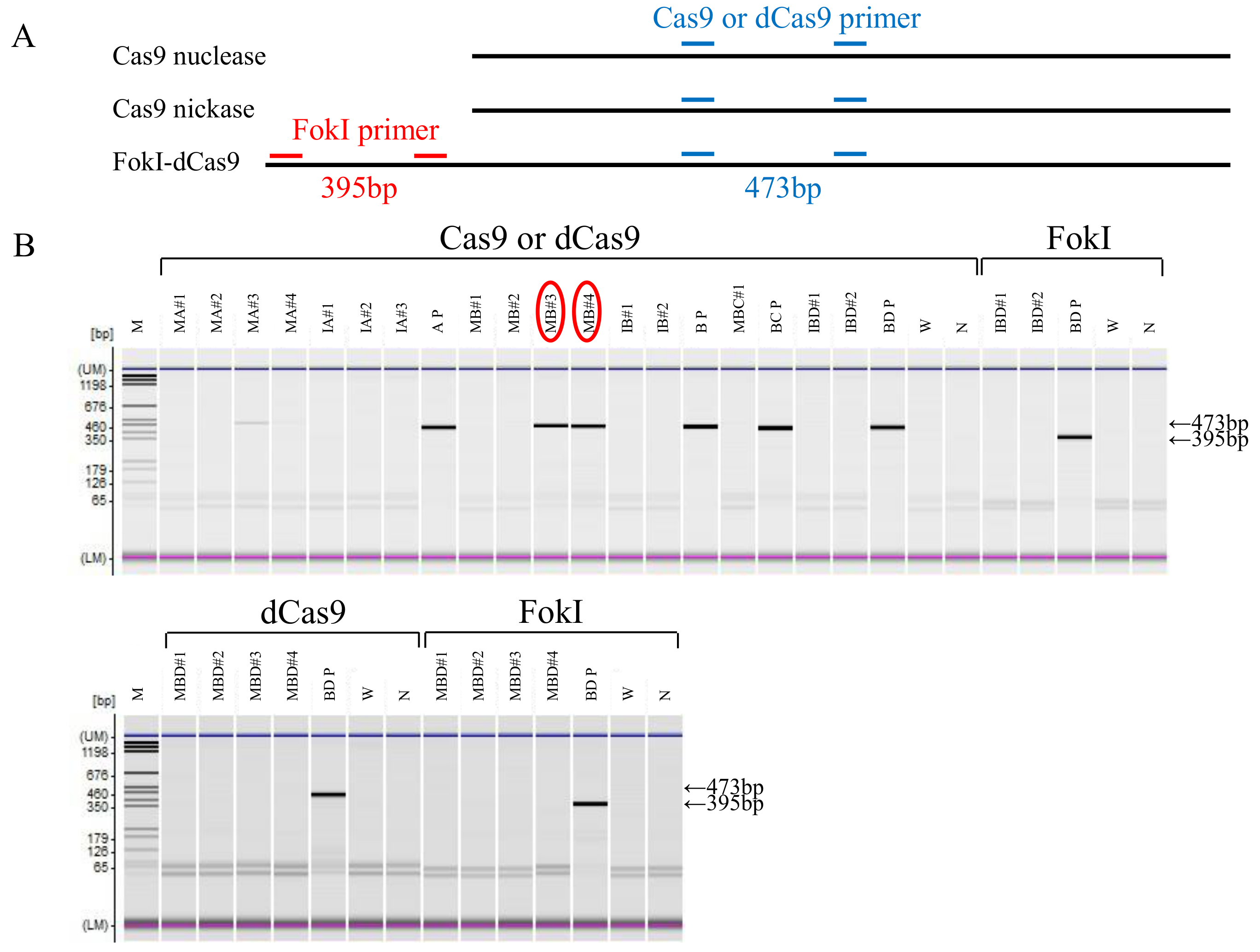

Supplement: Additional file 3: — Transgene analysis of founders. (A) Schematic drawing of the primers for each Cas9 variant. Red bars indicate primers for FokI detection. Blue bars indicate primers for Cas9 or dCas9 detection. The lengths of PCR products for FokI and Cas9/dCas9 are 395 and 473 bp, respectively. (B) Pseudo-gel images of transgene analysis. When vector DNA was integrated, a band of approximately 473-bp or 395-bp appears. Founder numbers shown on the upper side of each image are identical to Additional file 2. Red circles indicate transgene-positive founders. A P, B P, BC P, and BD P indicate positive controls for PCR amplification with Nulcease_A, Nuclease_B, Nickase_BC, and FokI-dCas9_BD plasmids, respectively, as templates. M, molecular weight maker (pGEM DNA Markers; Promega, Madison. WI, USA). W, negative control using founders without vector injection. N, negative control without template DNA. [file 12896_2015_144_MOESM3_ESM.tiff]
