# Supplementary material for: Production of knockout mice by DNA microinjection of various CRISPR/Cas9 vectors into freeze-thawed fertilized oocytes
Source: BMC Biotechnol. 2015 May 22;15:33. doi: 10.1186/s12896-015-0144-x (PMC4440308; doi:10.1186/s12896-015-0144-x)
Supplement: Additional file 4: — Top three candidates for potential off-target sites (5’-3’). [file 12896_2015_144_MOESM4_ESM.docx]

**Additional file 4. Top three candidates for potential off-target sites (5'-3')**

| Nuclease_A | TGCTTACAGAGATCACAGTCTAG AGCTGACCGAGAACACAGTCTAG TGCTGACCAAGATCACAGTCTGG | - |
| --- | --- | --- |
| Nuclease_B | CCAGCCCTAATGGCCAAGGTGGG CCAGTGCTCATGGGCAAGGTCGG ACAGAGTACATGGCCAAGGTAGG | - |
| Nickase_BC | CCAGCCCTAATGGCCAAGGTGGG CCAGTGCTCATGGGCAAGGTCGG ACAGAGTACATGGCCAAGGTAGG | GGAGCTGAGCAGGTGAGCGCGAG TCATCTTTGGAGGTGAGCGTAGG AGATGCCTGCAGGTGAGCGCTGG |
| FokI-dCas9_BD | CCAGCCCTAATGGCCAAGGTGGG CCAGTGCTCATGGGCAAGGTCGG ACAGAGTACATGGCCAAGGTAGG | CAGAGGACCTCTGGCTGATAAGG AAGGTAGGCGCTGGCTGATAAGG GAGGTGAGTGTTGGCTGATAGAG |
