# Supplementary material for: Production of knockout mice by DNA microinjection of various CRISPR/Cas9 vectors into freeze-thawed fertilized oocytes
Source: BMC Biotechnol. 2015 May 22;15:33. doi: 10.1186/s12896-015-0144-x (PMC4440308; doi:10.1186/s12896-015-0144-x)
Supplement: Additional file 5: — Primer sets for PCR. [file 12896_2015_144_MOESM5_ESM.docx]

**Additional file 5**. **Primer sets for PCR**

| Primers | Sequences (5'-3') | Purpose |
| --- | --- | --- |
| IL11F | TCTCTTGGGCACTTGACGAAG | Identification of IL11 mutant |
| IL11R | TACTCGAAGCCTTGTCAGCAC |  |
|  |  |  |
| A1F | ACCCTGCATTTTCTTCCCTC | Off-target analysis for gRNA A |
| A1R | CACAAGCCCCCTAAGTCAGA |  |
| A2F | TGGTCCTTGTCCTTCTAGGC |  |
| A2R | GGTGGTGGCTGAATGCTAAG |  |
| A3F | CAAGGGCACAGACTCACTCA |  |
| A3R | CAAACTCCCAAGGCTCAGTAA |  |
|  |  |  |
| B1F | AATGGCGGTTGACATTGTG | Off-target analysis for gRNA B |
| B1R | TGGGTGTCAGCTTCAGTGAG |  |
| B2F | CTTTCCCCTCCTCCAACAAG |  |
| B2R | CTCTCCTTGCGTTTCCATGTG |  |
| B3F | ATGTGATGCTGGGGGATTAC |  |
| B3R | CACTTATGGGTGGGAAAACG |  |
|  |  |  |
| C1F | GCACTGAGAAGGGTGGAAC | Off-target analysis for gRNA C |
| C1R | AAGTGTATGGAGGGGACAGC |  |
| C2F | GCTAAGGAAAGGGCAGTGAG |  |
| C2R | CATGGCACTTCGCACTATTAC |  |
| C3F | AGCTTTAACACCTTCCAGTGAC |  |
| C3R | GCAGCCTTGGACAGACTAAA |  |
|  |  |  |
| D1F | ACCCCATCCAAAAGTCAATG | Off-target analysis for gRNA D |
| D1R | CACTCCCCAAGAGAGGAAGA |  |
| D2F | GCCCTTGTGTAATGAAAGCAC |  |
| D2R | CTCACAGGCCAGGATACAGA |  |
| D3F | CCGACAATCCTTGCTCTGAC |  |
| D3R | CCTCCCCTGAGTTCCTTTTC |  |
|  |  |  |
| FokIF | TGCCTAAGAAGAAGCGGAAG | Identification of FokI |
| FokIR | CCACCACTCATTTGGATTGA |  |
|  |  |  |
| Cas9F | AAAGAGCGAGGAAACCATCA | Identification of Cas9 or dCas9 |
| Cas9R | GTGTCAGGGTCAGCACGATA |  |
|  |  |  |
| Reg3bF | TGCCCAGTGTTGGTAGAGTCA | Identification of Reg3b mutant |
| Reg3bR | TGGAAGACATAGGTAAGCAAGAGG |  |
|  |  |  |
| Reg3gF | TTCTGACGCTATCTGTTCTGGA | Identification of Reg3g mutant |
| Reg3gR | GGAGCCCACTTCTTTTGTGA |  |
|  |  |  |
| FokI-dCas9-ins-F | CCTAAGAAGAAGCGGAAGGTGAGCTCCCAACTCGTGAAGAGTGAACTTGAG | Construction of pX330A_FokI vectors |
| FokI-dCas9-ins-R | GGATCCACCGCCACCGAAATTGATCTCGCCATTGTTAAACTTGC |  |
| FokI-dCas9-vec-F | GGTGGCGGTGGATCCGACAAGAAGTACAGCATCGGCCTGG |  |
| FokI-dCas9-vec-R | CCGCTTCTTCTTAGGCATGGTGGCACCGGTCCAAC |  |
| FokI-dCas9-vec-R | CCGCTTCTTCTTAGGCATGGTGGCACCGGTCCAAC |  |
